# Therapeutic effect of intra-articular injected 3′-sialyllactose on a minipig model of rheumatoid arthritis induced by collagen

**DOI:** 10.1186/s42826-022-00119-2

**Published:** 2022-03-22

**Authors:** Young June Kim, Ju-young Lee, Mi-jin Yang, Hyun Jin Cho, Min-young Kim, Lila Kim, Jeong Ho Hwang

**Affiliations:** 1 Animal Model Research Group, Jeonbuk Branch Institute, Korea Institute of Toxicology, Jeollabukdo, 56212 Republic of Korea; 2 Pathology Research Group, Jeonbuk Branch Institute, Korea Institute of Toxicology, Jeollabukdo, 56212 Republic of Korea; 3 GeneChem Inc. A-501, 187 Techno 2-ro, Yuseong-gu, Daejeon, 34025 Republic of Korea

**Keywords:** Rheumatoid arthritis, 3′-Sialyllactose, Minipig, Intra-articular injection

## Abstract

**Background:**

Rheumatoid arthritis (RA) is a chronic inflammatory disease of joint, but there is no known cure.

3′-sialyllactose (3′-SL) is an oligosaccharide that is abundant in breast milk of mammals, and has anti-inflammatory properties. However, the efficacy of 3′-SL on RA remains unclear. The objective of the present study was to evaluate the therapeutic effect of 3′-SL after it was directly injected into the knee joint cavity of a RA minipig model.

**Results:**

Minipig RA model was induced by intra-articular injection of bovine type II collagen emulsified with complete or incomplete Freund’s adjuvant into left knee joint. In clinical assessment, lameness and swelling of the hindlimb and increased knee joint width were observed in all animals. After the onset of arthritis, 3′-SL (0, 2, 10, and 50 mg/kg) was directly administered to the left knee joint cavity once a week for 4 weeks. Compared to the vehicle control group, no significant difference in macroscopic observation of the synovial pathology or the expression of inflammation-related genes (*IL-1β*, *TNF-α*, and *COX2*) in the synovial membrane of the knee joint was found. In microscopic observation, cell cloning of the articular cartilage was significantly reduced in proportion to the concentration of 3′-SL administered.

**Conclusions:**

Our results suggest that intra-articular injected 3′-SL had a therapeutic effect on collagen-induced arthritis at the cellular level with potential as a medication for RA.

**Supplementary Information:**

The online version contains supplementary material available at 10.1186/s42826-022-00119-2.

## Background

Rheumatoid arthritis (RA) is a chronic inflammatory and autoimmune joint disease manifesting swelling, pain, and synovitis [1]. RA is characterized by progressive destruction of bone and synovial inflammation induction of articular cartilage. Synovial cell proliferation and neovascularization, pannus formation, infiltration of various inflammatory cells, and subsequent loss of chondral and bone matrix are commonly observed in histological findings [2, 3]. Excessive discharged inflammatory cytokines such as *IL-1b* and *TNF-a* play a key role in articular cartilage damage through cartilage matrix destruction and inflammation during RA pathogenesis [4, 5]. These cytokines can also induce the expression of *COX2* and *MMP*, causing degradation of articular cartilage and inflammation in collagen-induced arthritis models [6]. Thus, many anti-inflammatory drugs such as non-steroidal anti-inflammatory drugs (NSAIDs) and corticosteroids are used to manage patients with RA by regulating the inflammation pathway [7, 8]. However, these systemic drugs have diverse side effects on cardiovascular, gastric, and renal functions, thus limiting their long-term usages [9]. Intra-articular (IA) injection plays an important role in the treatment of joint diseases including rheumatism and osteoarthritis due to its safety with less chance of systemic exposure and undesired off-target effects [10]. Thus, IA injection is considered as an attractive alternative drug delivery route for RA management by maximizing therapeutic effects locally in the joint while limiting potential systemic adverse effects.

3′-Sialyllactose sodium salt (3′-SL), one of the most abundant oligosaccharides in human milk, exhibits anti-inflammatory properties and supports immune homeostasis [11]. Several attempts have been made to use 3′-SL as an alternative to NSAIDs known to have adverse effects [12]. Human clinical and animal studies have shown that 3′-SL, when administered by oral gavage, is safe for human consumption in food [13]. Furthermore, a recent study has shown that 3′-SL can ameliorate pathogenesis in an animal model of RA via blockade of the NK-kB signaling pathway, meaning that 3′-SL might have prophylactic and therapeutic effects on RA [14]. It has been concluded that 3′-SL could be a novel therapeutic drug for RA [14]. However, the efficacy of 3′-SL after it is administered into a lesion joint via IA injection has not been reported yet.

Pigs have been widely used in biomedical research because of its similarity in anatomy and physiology with human [15]. In addition, its relatively large size makes it easier to perform surgical procedures anatomically than small rodents. The objective of this study was to determine the efficacy of 3′-SL after it was directly injected into knee joints of minipigs with RA induced by injection of bovine collagen II (CII) emulsified with adjuvant into the knee joint cavity of minipigs. After direct injection of 3′-SL into the knee joint cavity, therapeutic effects of 3′-SL at different concentrations were evaluated based on clinical symptoms, gross observations of the knee joint, and histological findings.

## Results

### Effects of intra-articularly administrated 3′-SL on clinical manifestation of RA in a minipig model

**Fig. 1:**
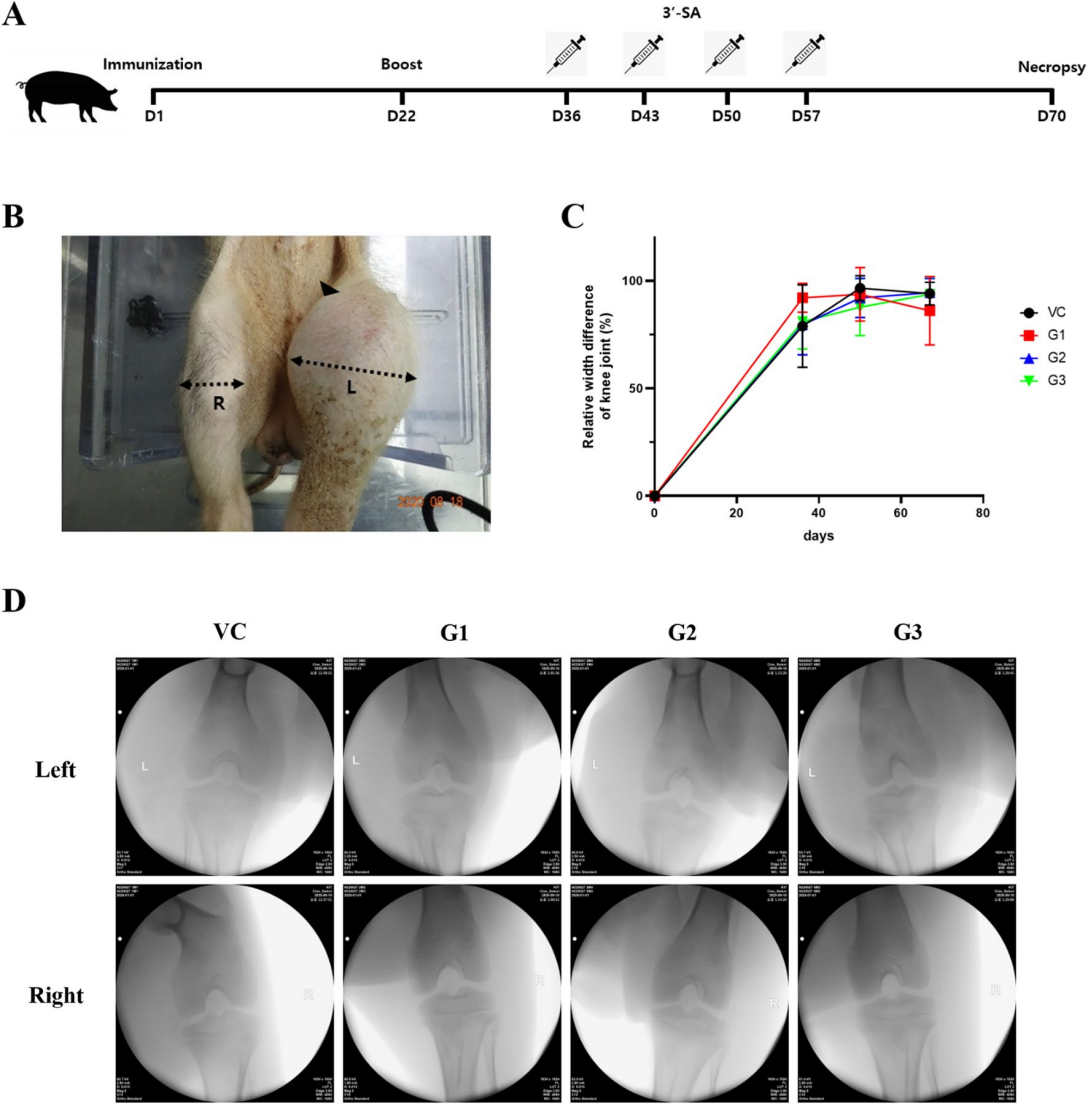
Effects of 3′-SL administration on clinical symptoms of a minipig model of rheumatoid arthritis induced by intra-articular injection of heterologous CII emulsified with CFA or IFA. **A** Experimental procedure for evaluating the therapeutic effect of 3′-SL in the rheumatoid arthritis minipig model. **B** Representative images of knee joints of rheumatoid arthritis-induced minipigs. Arrow head indicates swelling near the knee joint. Dotted arrow indicates the region where the width of the knee joint is measured. R: right knee joint; L: left knee joint. **C** Relative width difference of the knee joint. The difference in the width of the knee joint was calculated by subtracting the right knee value from the left knee value. These values were normalized to the length of D0. Error bars represent SD. **D** Representative C-arm images of the knee joint of a minipig model of rheumatoid arthritis. VC: vehicle control; G1: 2 mg/kg treatment group; G2: 10 mg/kg treatment group; G3: 50 mg/kg treatment group

Heterologous CII emulsified with complete Freund’s adjuvant (CFA) or incomplete Freund’s adjuvant (IFA) was injected into the knee joint cavity of each minipig to induce RA and the effect of 3′-SL administration on clinical symptoms related to RA was observed (Fig. 1A). After CII was injected into the knee joint, the width of the left knee joint was increased. There was no significant difference in the width of the knee joint between all groups treated with 3′-SL and the vehicle control group (Fig. 1C). Lameness and soft tissue swelling (= oedema) around the left knee joint were also observed (Fig. 1B). They were not ameliorated after intra-articular injection of 3′-SL. On radiography, no findings characteristic of RA such as osteopenia and erosion were observed (Fig. 1D). Collectively, these results indicated that RA in minipigs could be recapitulated by intra-articular injection of heterologous CII. However, RA-related clinical symptoms were not recovered by intra-articular administration of 3′-SL.

### Effects of intra-articularly administrated 3′-SL on knee joint of RA in a minipig model

**Fig. 2:**
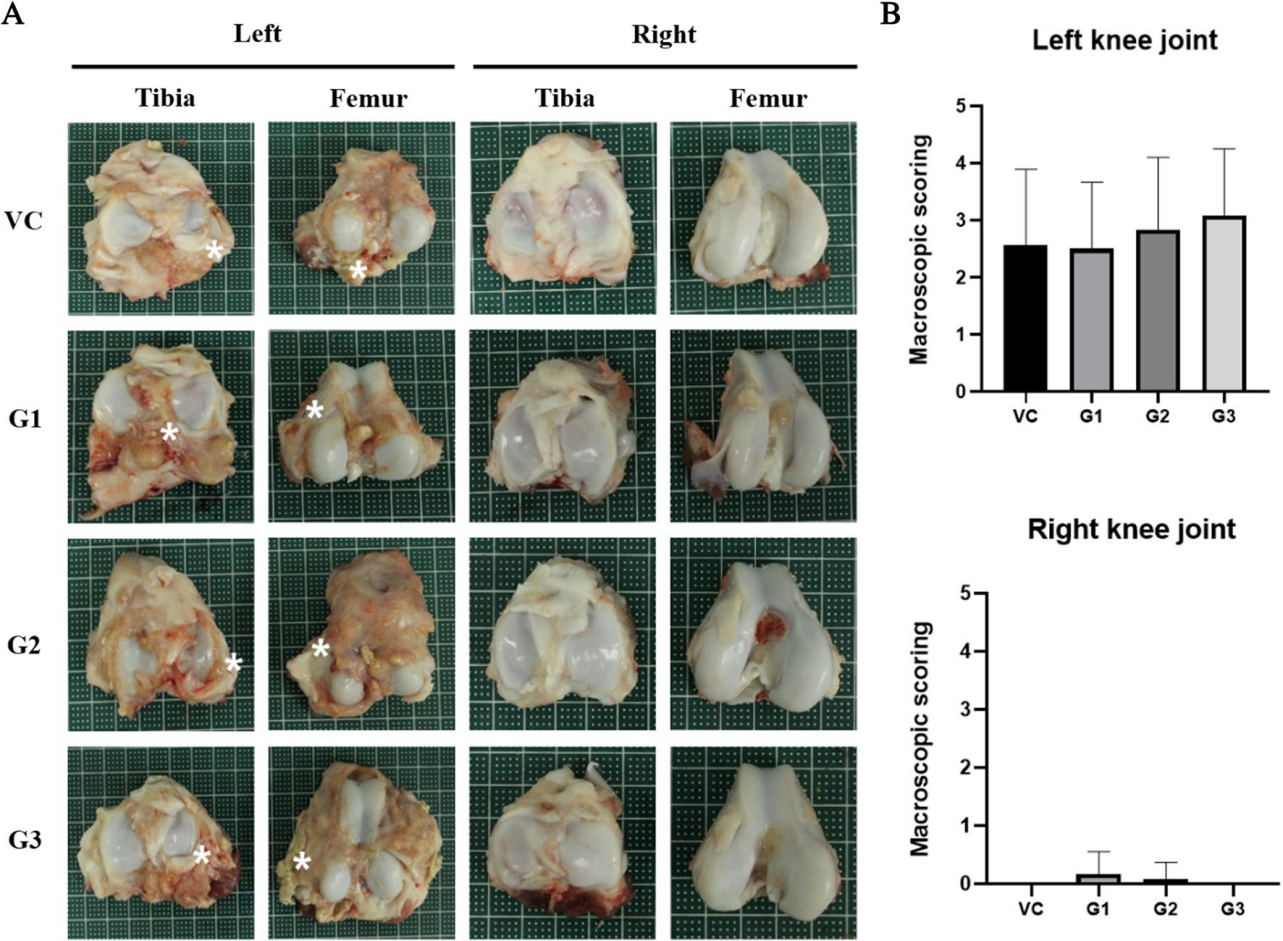
Effects of 3′-SL administration on rheumatoid arthritis in a minipig model based on macroscopic observations. **A** Representative images of the tibia and femur articular cartilage surface of rheumatoid arthritis in a minipig model. Asterisk indicates synovial hyperplasia. **B** Quantification results of macroscopic observations based on OARIS scores. Error bars represent SD. VC: vehicle control; G1: 2 mg/kg treatment group; G2: 10 mg/kg treatment group; G3: 50 mg/kg treatment group

We evaluated effects of 3′-SL on the knee joint of RA in a minipig model via gross and microscopic observations. At necropsy, no abnormalities caused by direct injection of 3′-SL into the knee joint were observed in gross observation. No lesions on cartilage that could be made by syringe during the intra-articular injection process were observed. Like radiation results, no findings related to RA were observed in the cartilage or bone of the knee joint injected with 3′-SL. Compared with the right knee joint, a proliferation of soft tissue around the left knee joint was found (Fig. 2), which recapitulated synovial membrane hyperplasia, a typical symptom of RA [16]. We quantified cartilage and synovial pathology by macroscopic scoring of the knee joint using Osteoarthritis Research Society International (OARSI) score and found no significant difference between the vehicle control and treat groups (Fig. 2B).

**Fig. 3:**
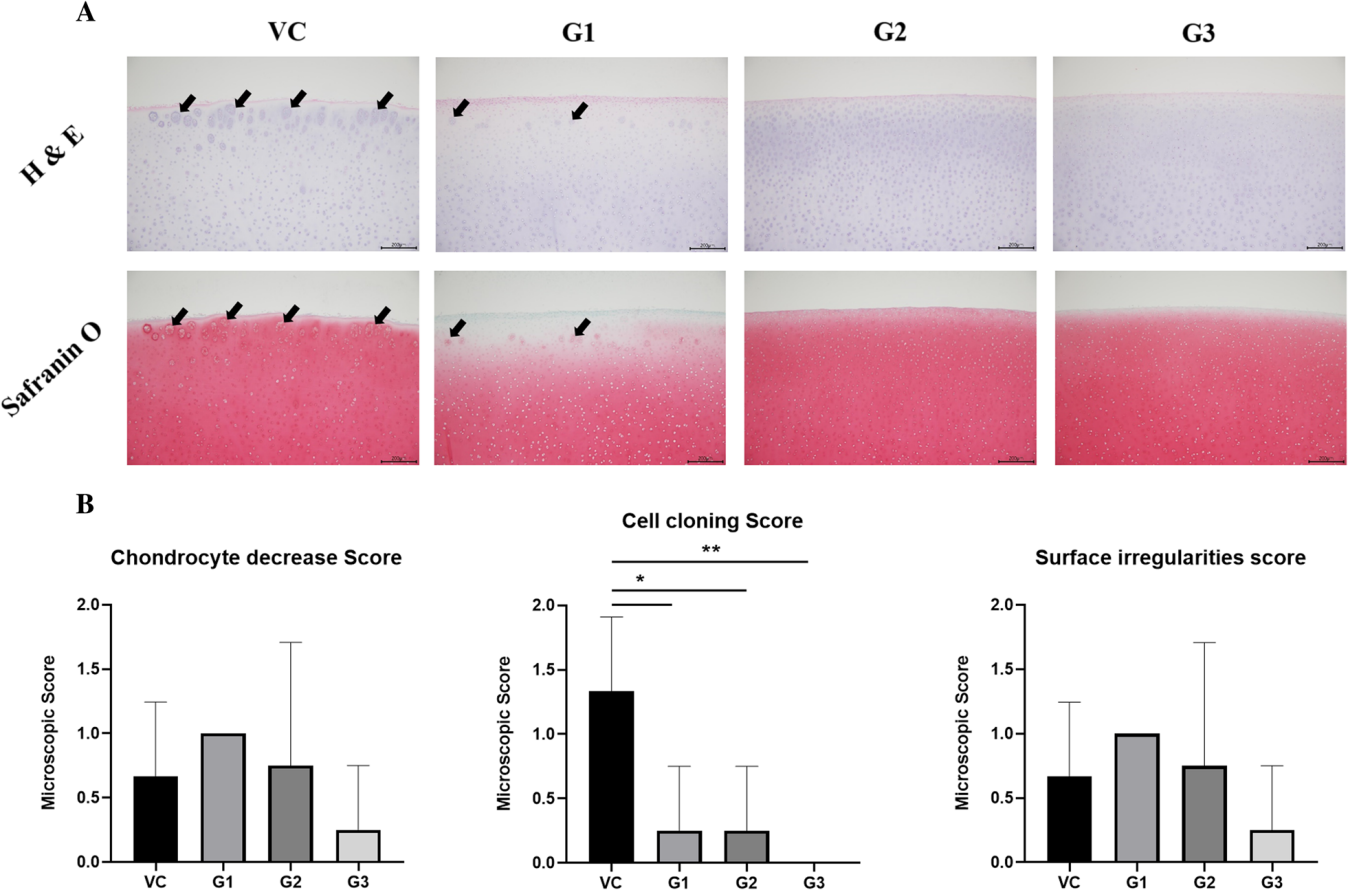
Effects of 3′-SL administration on rheumatoid arthritis based on microscopic observation of rheumatoid arthritis induced minipig model. **A** Representative cell cloning images obtained by H&E and Safranin-O staining of the articular cartilage of the knee joint. Black arrow indicates cell cloning. 100 × magnification, Scale bar = 200 µm. **B** Quantification results of chondrocyte density, cell cloning, and surface irregularities via macroscopic observations based on OARIS scores. Error bars represent SD. *, *p* < 0.05; **, *p* < 0.01, One-way analysis of variance. VC: vehicle control; G1: 2 mg/kg treatment group; G2: 10 mg/kg treatment group; G3: 50 mg/kg treatment group

Next, we quantified cartilage disruption by microscopic scoring of articular cartilage such as chondrocyte decrease, cell cloning, and surface irregularities on both femur and tibia plateau cartilage. There was no significant difference in chondrocyte density or articular surface (Fig. 3A, C). Interestingly, cell cloning of the articular cartilage was significantly reduced in proportion to the concentration of 3′-SL administered (*p* < 0.005, Fig. 3B). These results indicated that cartilage disruption could be partially recovered by intra-articular administration of 3′-SL.

### Effects of 3′-SL on the expression of pathogenic genes related to RA in synovial membrane of RA in a minipig model

**Fig. 4:**
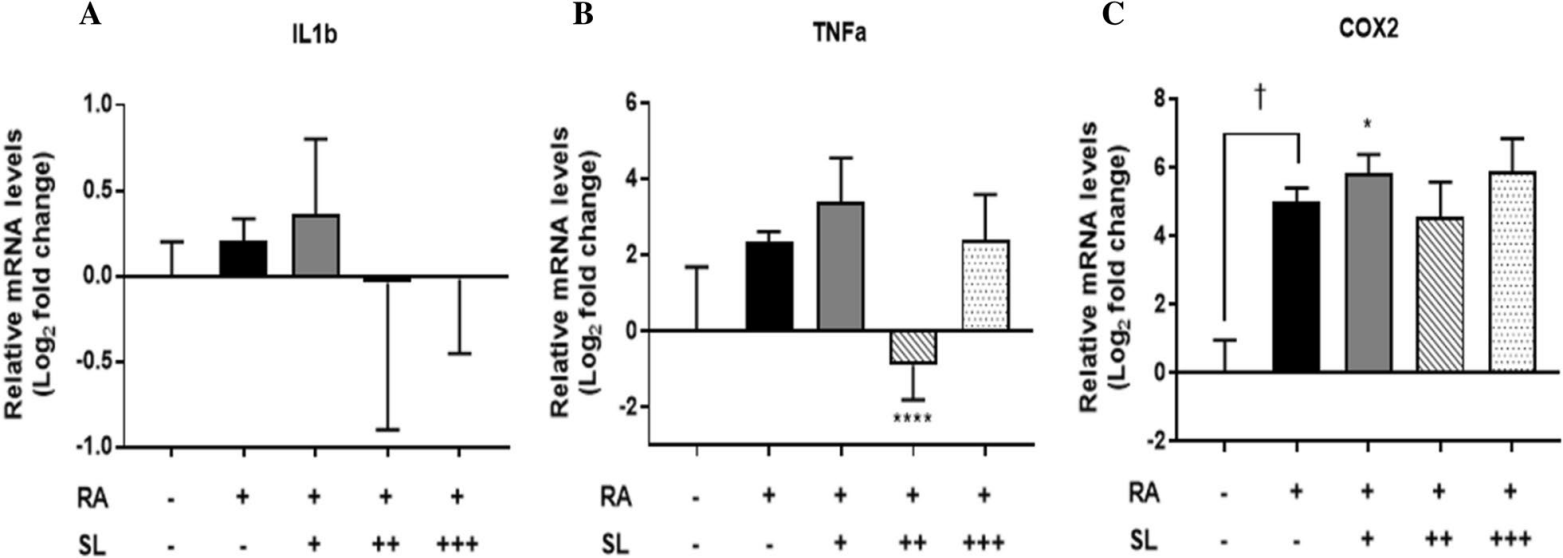
Effects of 3′-SL administration on rheumatoid arthritis related pathogenic pathway in the synovial membrane of a minipig model of RA. **A**–**C** Relative expression levels of IL-1β, TNFα, and COX2 in the synovial membrane of rheumatoid arthritis induced knee joints by qRT-PCR. Error bars represent SD. *, *p* < 0.05; ****, *p* < 0.001, One-way analysis of variance. VC: vehicle control; G1: 2 mg/kg treatment group; G2: 10 mg/kg treatment group; G3: 50 mg/kg treatment group

To investigate the effect of intra-articularly injected 3′-SL on genes associated with RA pathogenesis in a synovial membrane of RA-induced knee joint, we analyzed the expression of RA pathogenesis (*IL-1β* and *TNFα*) and inflammatory-related (*COX2*) genes. First, gene expression levels in synovial membranes of left knees of minipigs with induced RA were compared to those of right knees as controls. Compared to the right knee, mRNA expression levels of *IL-1β* and *TNFα* in the synovial membrane of the left knee were increased by RA induction, although such increases were not not statistically significant (Fig. 4A, B). On the other hand, *COX2* mRNA level was significantly (*p* < 0.005) increased by intra-articular injection of heterologous CII (Fig. 4C).

Next, expression levels of *IL-1β*, *TNFα*, and *COX2* mRNAs in synovial membranes of knee joints of RA-induced minipigs after treatment with 3′-SL at different concentrations were determined. No significant change in gene expression of *IL-2* was observed between groups treated with 3′-SL. *TNFα* mRNA level was significantly (*p* < 0.0001) reduced in the 10 mg/kg treatment group. The expression of *COX2* mRNA was significantly (*p* < 0.05) increased in the 2 mg/kg treatment group. These data indicated that the inflammatory pathway was activated by collagen-induced RA. However, intra-articular injection of 3′-SL did not have anti-inflammatory effects in the synovial membrane.

## Discussion

In this study, we investigated the efficacy of intra-articular injected 3′-SL into the knee joint of a minipig with RA induced by injection of heterologous CII emulsified with adjuvant into the knee joint. RA-related symptoms including lameness, swelling, synovial hyperplasia, and cartilage disruption were observed after RA induction. In addition, expression level of *COX2* as an inflammatory-related gene was significantly increased in the synovial membrane of RA-induced knee joint. After intra-articular injection of 3′-SL, cartilage lesions of the knee joint were recovered at the cellular level. Our study highlighted that a minipig RA model could be used for efficacy evaluation. The efficacy of intra-articular injection instead of oral administration of 3′-SL was evaluated.

In present study, we established RA model induced by intra-articular injection of a bovine CII in minipigs. The advantage of minipigs as an RA model compared to other animals is that minipigs have functional similarities such as bone mineral density, remodeling rate, and healing with those of human as well as structural similarities such as intra-articular cartilage and associated ligaments in synovial joints [17]. RA is a representative autoimmune and inflammatory disease that primarily affects joints, and 3′-SL regulates the inflammatory response. Thus the immune system of laboratory animals plays a key role in this study. Previous studies have shown that pigs have a relatively similar immune system to humans compared to small rodents [18]. Therefore, minipigs would have a relative advantage in reproducing rheumatoid arthritis with human-like symptoms and evaluating the efficacy of 3′-SL.

Collagen induced arthritis (CIA) rodent models have been fabulously used in the preclinical research of RA. In the present study, RA was successfully induced by intra-articular injection of heterologous CII, consistent with a previous study [19]. However, although physical alterations such as the abnormal proliferation of soft tissue and claudication caused by RA were observed, we found no radiological abnormalities related to RA in the present study. Chronic inflammation in RA accelerates bone loss and inhibits bone regeneration, leading to osteopenia [20]. In the previous pig RA model, symptoms such as joint deformities and dislocations were observed, but osteopenia and erosion were not observed [19]. The reason for the failure to reproduce osteopenia in minipig RA model may be due to insufficient time for RA induction. The time for RA induction in this study was approximately 35 days, which was sufficient time to induce an inflammatory response in the joint, but it might be not enough to induce bone-related symptoms. Also, considering that, in mouse, CIA can be induced only in susceptible strain such as DBA/1, B10 [21], further studies will be required to induce a more sophisticated RA model in minipigs.

It has been previously reported that symptoms of CIA in mice might slightly differ depending on the strain used in the experiment [22]. Thus, we believe that such differences between our study and the previous study might be caused by different strains of minipig used. Our method could be applied to evaluate the effectiveness of various intra-articular injection drugs.

Pro-inflammatory cytokines such as *IL-1β* and *TNFα* secreted from activated macrophages and neutrophils can amplify systemic inflammatory responses [23]. These cytokines are directly involved in the disruption of homeostasis in the articular joint and cartilage. When chondrocytes secrete more inflammatory cytokines, they can amplify injurious cellular responses. In this study, we found no increment in the expression of pro-inflammatory cytokines after RA induction.

However, mRNA level of *COX2*, an inflammatory cytokine, was significantly increased after RA induction, indicating that the arthritis induced by heterologous CII was primarily associated with acute inflammatory responses rather than rheumatoid arthritis [24]. These pathological features are similar to those occurring in a rodent model, but not those in humans[16]. Therefore, more sophisticated methods for modeling RA in animals are necessary.

In this study, the anti-inflammatory effects of 3′-SL injected into synovial cavity was not statistically significant. Although it was confirmed that the pathological findings were partially improved at the cellular level, the efficacy according to the administered dose of 3′-SL on the inflammatory factor could not be confirmed. This was an unexpected result that was in contrast to the results of previous studies [14, 25]. Unlike a method of the previous studies in which the test substance 3′-SL was taken orally, 3′-SL was injected directly into the joint cavity in this study. In order to evaluate the efficacy of 3′-SL as a therapeutic agent, the administration method was modified from oral administration to intra-articular injection, which is a method commonly used in orthopedics [26]. These results indicate that the efficacy of 3′-SL might be compromised when 3′-SL is administered directly to the site of inflammation.

## Conclusions

We have shown that 3′-SL injected directly into the joint had an alleviation effect at the cellular level against CIA in minipigs. Therefore, 3′-SL would be considered to have potential to be a medication for RA through a novel route of administration.

## Methods

### Ethics statement

All experimental protocols were approved by the Animal Care and Use Committee of the Korea Institute of Toxicology (KIT) and complied with the Association for Assessment and Accreditation of Laboratory Animal Care International Animal Care Policies (Approval No. 2006-0200).

### Mini-pig rheumatoid arthritis model

Rheumatoid arthritis was induced according to a previous study [19] with minor modifications. Briefly, a total of 15 specific pathogen free (SPF) minipigs (3 to 4 months old males with body weight of 15–20 kg) were obtained from OPTIPHARM (OPTIPHARM Co., Ltd., Korea). All animals were sedated with an intramuscular injection of ketamine (20 mg/kg) and xylazine (2.5 mg/kg). Anesthesia was performed by isoflurane inhalation. For all animals, left knee joint cavities were directly administered with a mixture of 0.5 mg/kg bovine type II collagen (CII; Chondrex, WA, USA) emulsified with an equivalent volume of 2 mg/ml CFA (Chondrex) intra-articularly on Day 1. The second immunization was performed on Day 22 using the same procedure except that IFA (Chondrex) instead of CFA was used.

### Intraarticular injection of 3′-SL

Fifteen SPF minipigs were randomly divided into four groups: vehicle control (0 mg/kg, n = 3), G1 (2 mg/kg, n = 4), G2 (10 mg/kg, n = 4), and G3 (50 mg/kg, n = 4). In a previous study using RA mouse model [14], the efficacy of 3′-SL was observed at 100 mg/kg and 500 mg/kg treat groups. In consideration of the human clinical trial dose and the dose of the 3′-SL formulation [27], the administration dose of 3′-SL was set to 2 mg/kg for low doses, 10 mg/kg for middle doses, and 50 mg/kg for high doses. A control group administered with placebo was also established. 3′-SL was provided by GeneChem Inc (GeneChem Inc., Daejeon, Korea) and dissolved in 0.9% normal saline. All animals were sedated with an intramuscular injection of ketamine (20 mg/kg) and xylazine (2.5 mg/kg). Anesthesia was maintained by isoflurane inhalation. For all groups, 3′-SL was directly injected with a syringe with 21G into left knee joint cavities once a week for 4 weeks (Day 36, 43, 50, and 57).

### Gross observation, clinical evaluation, and radiological assessment

Widths of both knee joints were measured in triplicate with a Vernier caliper ruler at the widest region of the knee joint before the first injection of CII and on Day 36, Day 50, and Day 67. The difference in the width of knee joint was calculated by subtracting the right knee value from the left knee value. Knee joints of all animals were scanned for morphological changes on Day 67.

### Scoring of cartilage destruction and histochemistry

Macroscopic scoring was performed for the synovium and cartilage as previously described [28, 29]. Gross observations at the time of sacrifice were performed for each knee joint. The histopathology of articular cartilage was assessed according to the following criteria by one of us without prior knowledge of experimental groups; Grade 0—No definite synovitis, Grade 1—Define synovitis, Grade 2—Severe exudative and proliferative synovitis, pannus and erosive change present, and Grade 3—All of the above with marked joint disorganization. Materials for histological analysis were fixed in 10% neutral-buffered formalin and decalcified with 10% formic acid for 4 weeks. After decalcification, knee joint cartilages were embedded in paraffin and sectioned at a thickness of 4 μm. H&E staining and Safranin-O staining were performed for tibia and femur cartilage sections and scored using OARSI guidelines [28, 29].

### Quantitative reverse transcription–polymerase chain reaction (qRT-PCR)

Total RNA was isolated from the synovial membrane using TRIzol (15596018, Thermo Fisher Scientific, MA, USA) and cDNA was synthesized with 1 μg RNA using a Quantitect Reverse Transcription Kit (205313, Qiagen, Hilden, Germany) according to the manufacturer’s instructions. qRT-PCR was performed using a Power SYBR^™^ Green PCR Master Mix (4368702, Applied Biosystems, CA, USA) and porcine primers for GAPDH, IL2-β, TNF-α, and COX2. Primer sequences used for qRT-PCR are listed in Additional file 1. qRT-PCR conditions were as follows: 95 °C for 10 min, 40 cycles of 95 °C for 15 s and 60 °C for 1 min on a PCR machine (A28134, Applied Biosystems). mRNA levels of target genes were normalized to the expression of GAPDH (ΔC_t_ = C_t_ gene of interest − C_t_ GAPDH) and described as relative mRNA expression (2^△Ct sample−△Ct control^) or fold-change.

### Statistical analysis

The dataset was statistically analyzed using GraphPad Prism 8 (GraphPad Software, CA, USA). All results are presented as mean ± SD. Statistical significance for each experiment was evaluated using one-way analysis of variance (ANOVA) and Turkey’s multiple comparison test (Duncan’s multiple range test). *p* values < 0.05 were considered significant.

## Supplementary Information

**Additional file 1.** List of rimers used for qRT-PCR.

## Data Availability

All experiment data during this study are included in this manuscript and additional files.

## Abbreviations

RA: Rheumatoid arthritis
3′-SL: 3′-Sialyllactose
IL-2 *β*: Interleukin 2 beta
TNF-*α*: Tumor necrosis factor-alpha
COX2: Cyclooxygenase 2
MMP: Matrix metalloproteinase
NSAIDs: Non-steroidal anti-inflammatory drugs
IA: Intra articular
NF-kB: Nuclear factor kappa light chain enhancer of activated B cells
CII: Collagen II
CFA: Complete Freund’s adjuvant
IFA: Incomplete Freund’s adjuvant
OARSI: Osteoarthritis Research Society International
CIA: Collagen induced arthritis
SPF: Specific pathogen free
