# Supplementary figures and images for: Therapeutic effect of intra-articular injected 3′-sialyllactose on a minipig model of rheumatoid arthritis induced by collagen
Source: Lab Anim Res. 2022 Mar 22;38:8. doi: 10.1186/s42826-022-00119-2 (PMC8939226; doi:10.1186/s42826-022-00119-2)

Supplementary Material 1.


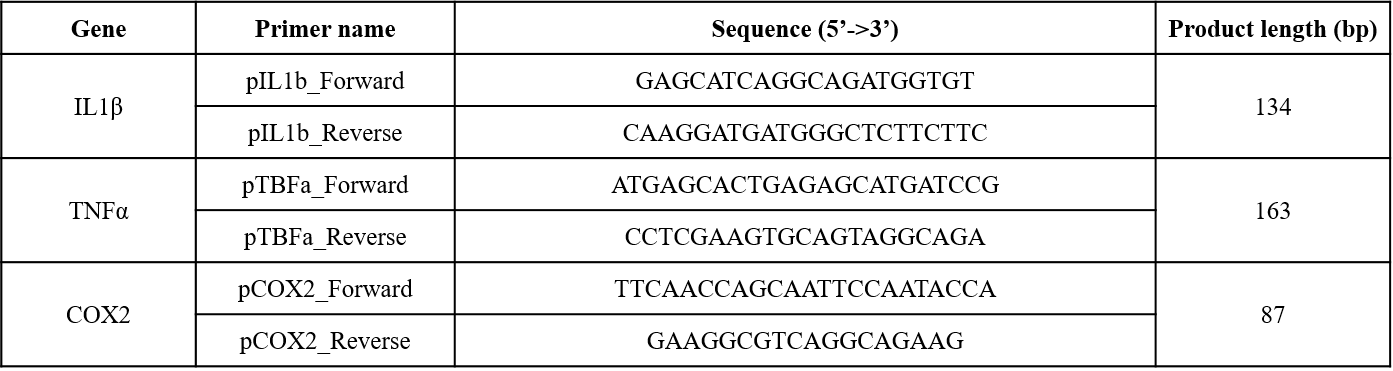

Supplement: Supplementary file 1 — Additional file 1. List of rimers used for qRT-PCR. [file 42826_2022_119_MOESM1_ESM.docx]
